# Genomic and immune characteristics of *HER2*‐mutated non‐small‐cell lung cancer and response to immune checkpoint inhibitor‐based therapy

**DOI:** 10.1002/1878-0261.13439

**Published:** 2023-04-29

**Authors:** Hai‐Yan Tu, Kai Yin, Xiaotian Zhao, E‐E Ke, Si‐Pei Wu, Yang‐Si Li, Mei‐Mei Zheng, Si‐Yang Maggie Liu, Chong‐Rui Xu, Yue‐Li Sun, Jia‐Xin Lin, Xiao‐Yan Bai, Yi‐Chen Zhang, Qing Zhou, Jin‐Ji Yang, Wen‐Zhao Zhong, Bing‐Chao Wang, Xu‐Chao Zhang, Dongqin Zhu, Lingling Yang, Qiuxiang Ou, Yi‐Long Wu

**Affiliations:** ^1^ Guangdong Lung Cancer Institute, Guangdong Provincial People's Hospital (Guangdong Academy of Medical Sciences) Southern Medical University Guangzhou China; ^2^ The Second School of Clinical Medicine Southern Medical University Guangzhou China; ^3^ Geneseeq Research Institute Nanjing Geneseeq Technology Inc. China; ^4^ Guangdong Lung Cancer Institute, Medical Research Center Guangdong Provincial People's Hospital, Guangdong Academy of Medical Sciences Guangzhou China; ^5^ Guangdong Provincial Key Laboratory of Translational Medicine in Lung Cancer Guangdong Provincial People's Hospital, Guangdong Academy of Medical Sciences Guangzhou China; ^6^ Department of Hematology, First Affiliated Hospital, Institute of Hematology, School of Medicine, Key Laboratory for Regenerative Medicine of Ministry of Education Jinan University Guangzhou China; ^7^ Chinese Thoracic Oncology Group (CTONG) Guangzhou China

**Keywords:** advanced NSCLC, exon 20 insertion, human epidermal growth factor receptor 2, immune checkpoint inhibitor, non‐exon 20 insertion

## Abstract

The efficacy of immunotherapy in advanced *HER2*‐mutated non‐small‐cell lung cancer (NSCLC) remains incomprehensively studied. A total of 107 NSCLC patients with *de novo HER2* mutations were retrospectively studied at Guangdong Lung Cancer Institute [GLCI cohort, exon 20 insertions (ex20ins): 71.0%] to compare clinical/molecular features and immune checkpoint inhibitor (ICI)‐based therapy efficacy between patients with ex20ins and non‐ex20ins. Two external cohorts (TCGA, *n* = 21; META‐ICI, *n* = 30) were used for validation. In the GLCI cohort, 68.2% of patients displayed programmed death‐ligand 1 (PD‐L1) expression < 1%. Compared with ex20ins patients, non‐ex20ins patients had more concurrent mutations in the GLCI cohort (*P* < 0.01) and a higher tumour mutation burden in the TCGA cohort (*P* = 0.03). Under ICI‐based therapy, advanced NSCLC patients with non‐ex20ins had potentially superior progression‐free survival [median: 13.0 *vs*. 3.6 months, adjusted hazard ratio (HR): 0.31, 95% confidence interval (CI): 0.11–0.83] and overall survival (median: 27.5 *vs.* 8.1 months, adjusted HR: 0.39, 95% CI: 0.13–1.18) to ex20ins patients, consistent with findings in the META‐ICI cohort. ICI‐based therapy may serve as an option for advanced *HER2*‐mutated NSCLC, with potentially better efficacy in non‐ex20ins patients. Further investigations are warranted in clinical practice.

## Graphical abstract

*HER2*‐mutated non‐small‐cell lung cancer (NSCLC) patients with *HER2* mutations other than exon 20 insertions (non‐ex20ins) had higher tumour mutation burden than patients with *HER2* ex20ins, whereas similar programmed death‐ligand 1 expression was observed. Under immune checkpoint inhibitor‐based therapy, advanced NSCLC patients with *HER2* non‐ex20ins had potentially superior progression‐free survival and overall survival compared with patients with *HER2* ex20ins.

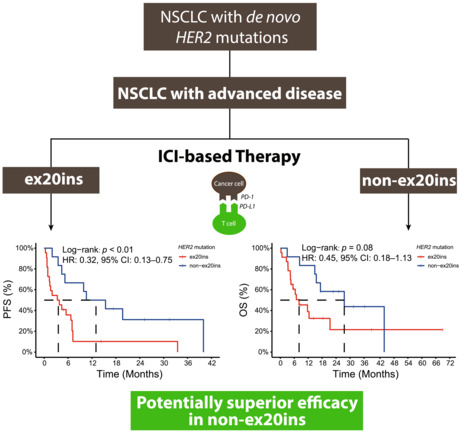

**Abbreviations**
CI: confidence interval
ex20ins: exon 20 insertions
FFPE: formalin‐fixed paraffin‐embedded
GLCI: Guangdong Lung Cancer Institute
*HER2*
: human epidermal growth factor receptor 2
HR: hazard ratio
ICI: immune checkpoint inhibitor
IHC: immunohistochemistry
NGS: next‐generation sequencing
non‐ex20ins: mutations other than ex20ins
NSCLC: non‐small‐cell lung cancer
ORR: objective response rate
OS/mOS: overall survival/median overall survival
PD‐1: programmed cell death‐receptor 1
PD‐L1: programmed cell death‐ligand 1
PFS/mPFS: progression‐free survival/median progression‐free survival
TCGA: The Cancer Genome Atlas Program
T‐DM1: ado‐trastuzumab emtansine
T‐DXd: trastuzumab deruxtecan
TKI: tyrosine kinase inhibitor
TMB: tumour mutational burden
TME: tumour microenvironment
TPS: tumour proportion score

## 1 Introduction

Human epidermal growth factor receptor 2 (*HER2/ERBB2*) is an oncogenic driver of tumour cell proliferation and metastasis [1]. Mutated *HER2* genes are rare (prevalence: 2–4%) in non‐small‐cell lung cancer (NSCLC) patients, and are enriched in patients who are females, non‐smokers and younger, and in adenocarcinoma patients [2, 3]. Over 20 types of *HER2* exon 20 insertion (ex20ins) mutations have been identified in NSCLC, accounting for 25–50% of NSCLC patients exhibiting *HER2* mutations [4, 5, 6]. However, the genomic and immune characteristics of patients with different *HER2* mutations have not been comprehensively investigated.

Advanced NSCLC patients harbouring *HER2* mutations have a worse prognosis (median survival: 1.9–2.3 years) [3, 7], compared with those harbouring *EGFR* or *ALK* mutations. Treatments targeting *HER2* mutations in advanced NSCLC include tyrosine kinase inhibitors (TKIs) and *HER2*‐antibody‐drug conjugates (ADCs). Objective response rates (ORRs) of treatment with TKIs, such as afatinib [8], dacomitinib [9], or pyrotinib [10, 11], are < 30%. The efficacy of TKIs in NSCLC treatment may depend on *HER2* mutation subtypes [12, 13, 14]; however, no significant differences in pyrotinib effectiveness were found between patients with *HER2* ex20ins and non‐ex20ins mutations. Under ADC treatment, patients receiving ado‐trastuzumab emtansine (T‐DM1) and trastuzumab deruxtecan (T‐DXd) achieved ORRs of 50% and 55%, respectively, and the median progression‐free survival (mPFS) of T‐DXd treatment was 8.2 months [15, 16]. However, the majority of patients in these studies harboured *HER2* ex20ins mutations (73–86%), and there was a scarcity of data on patients carrying *HER2* non‐ex20ins mutations [17]. The toxicity of T‐DXd remains an important issue; interstitial lung disease is presented by 26% of T‐DXd users [15], despite the recent approval (by the U.S. Food and Drug Administration) of T‐DXd for patients with unresectable or metastatic *HER2*‐mutated NSCLC. Overall, there is a need to identify effective and tolerable treatments for patients with advanced *HER2*‐mutated NSCLC.

Because neither TKIs nor ADCs have been approved as first‐line treatments for *HER2*‐mutated NSCLC patients with advanced disease [18], immune checkpoint inhibitors (ICIs) with/without platinum doublet chemotherapy serve as the standard first‐line therapy [19]. Treatment‐naive patients receiving ICI combination treatment achieved ORR of 52% and mPFS of 6 months [20], whereas other studies have reported ORRs of 7.0–27.0% and mPFS of 2.2–4.0 months under ICI‐based therapy in second‐line or subsequent treatment [21, 22]. Notably, it has been revealed that none of the ICI responders harboured *HER2* YVMA mutation [23], suggesting that ICI efficacy in patients with *HER2* ex20ins mutations might differ from patients harbouring other *HER2* mutations. Moreover, the efficacy of immunotherapy has not been systematically investigated in *HER2* non‐ex20ins patients, owing to the low prevalence of *HER2* mutations in NSCLC and the focus on *HER2* ex20ins. Thus, the association between *HER2* mutation subtypes and immunotherapy efficacy remains controversial, and the potential underlying mechanisms have not been comprehensively investigated.

This study aimed to compare the molecular features and tumour microenvironment (TME) characteristics of NSCLC patients harbouring *HER2* ex20ins and *HER2* non‐ex20ins mutations. The efficacy of ICI‐based therapy was analysed in patients with advanced disease. Two external datasets of *HER2*‐mutated NSCLC patients were used to validate the results.

## 2 Materials and methods

### 2.1 Patients

This retrospective study was performed at Guangdong Lung Cancer Institute (GLCI), Guangdong Provincial People's Hospital; participants in the GLCI cohort were consecutively enrolled between January 2016 and December 2020. The main inclusion criteria were follows: (1) adults having at least 18 years of age; (2) pathologically confirmed NSCLC, according to the 2016 World Health Organization classification; and (3) identified with somatic *de novo HER2* mutations in tissue or plasma samples. Patients with other oncogenic drivers, including sensitizing *EGFR* mutations, *BRAF* ^V600E^ mutation, *KRAS* ^G12X/Q61H^ mutation, *ALK*/*NTRK*/*RET*/*ROS1* fusion, and *MET* exon 14 skipping, were excluded. Insertion mutations in *HER2* exon 20 (residues 770–831) were identified as *HER2* ex20ins; *HER2* mutations outside exon 20 (residues before 770 or after 831) were identified as *HER2* non‐ex20ins mutations. Patients harbouring both *HER2* exon 20 and non‐ex20ins mutations were grouped into the non‐ex20ins subgroup. The clinical stages and histological subtypes of NSCLC were determined according to the ‘8th edition of the American Joint Committee on Cancer classification system’. Demographics and clinical characteristics of the participants, including age, sex, Eastern Cooperative Oncology Group performance status, and smoking history were obtained from the electronic medical record system of Guangdong Provincial People's Hospital.

**Fig. 1:**
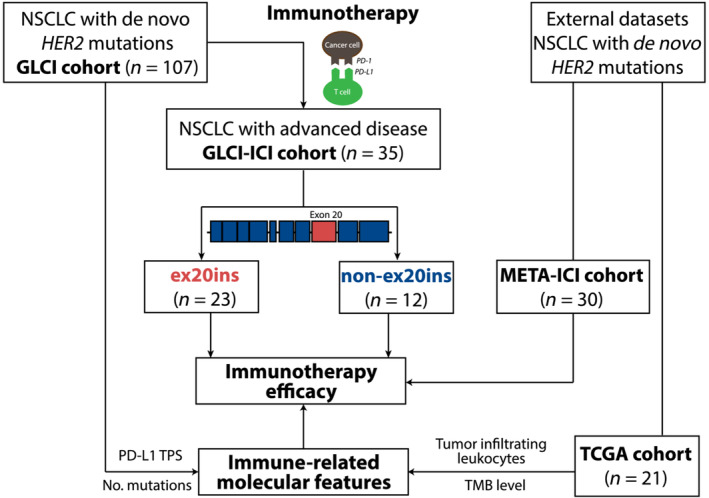
The flow chart of patient enrolment and validation cohorts. A total of 107 NSCLC patients harbouring *HER2* mutations were enrolled in the GLCI cohort, including 76 patients with *HER2* ex20ins and 31 patients with *HER2* non‐ex20ins mutations. The efficacy of ICI‐based therapy and TME features were investigated among 35 of these 107 patients (GLCI‐ICI cohort). Two external datasets of *HER2*‐mutated NSCLC patients, The Cancer Genome Atlas Program (TCGA, *n* = 21) and META‐ICI (*n* = 30) were used for result validation.

ICI‐based therapy was defined as a regimen that included inhibitors of programmed cell death‐1 receptor or its ligand (PD‐1/PD‐L1). Advanced NSCLC patients in the GLCI cohort treated with ICI monotherapy or ICI combination therapy were grouped as the GLCI‐ICI cohort (Fig. 1), and they were followed up until November 2021 or until death. The clinical response to ICI‐based therapy was evaluated using computed tomography according to the ‘Response Evaluation Criteria in Solid Tumours version 1.1’. The study procedures were approved by the Ethics Committee of Guangdong Provincial People's Hospital (2013185H), and written informed consent was obtained from each patient. The study methodologies also conformed to the standards set by the Declaration of Helsinki.

### 2.2 External cohorts

From The Cancer Genome Atlas Program (TCGA), NSCLC patients with *de novo HER2* mutations and without other NSCLC oncogenic drivers were included in the TCGA cohort for the validation of genomic features and immune characteristics (Fig. 1). Tumour mutational burden (TMB) of each patient in this cohort was recalculated using a standardized method [24]. The abundance of tumour‐infiltrating leukocytes was estimated by CIBERSORT using RNA‐Seq data, and significantly differentially expressed genes between patients with *HER2* ex20ins and *HER2* non‐ex20ins mutations were investigated using Gene Set Enrichment Analysis.

From six external data sets, advanced *HER2*‐mutated NSCLC patients who did not harbour other NSCLC oncogenic drivers and received ICI‐based therapy were grouped into the META‐ICI cohort (Fig. 1), including 10 patients from a study by Rizvi et al. [25], nine patients from a study by Gandara et al. [26], two patients from a study by Miao et al. [27], five patients from a study by Anagnostou et al. [28], and four patients from a study by Samstein et al [29]. Clinicopathological and prognostic data were analysed.

### 2.3 DNA extraction, library preparation, and next‐generation sequencing data processing

Genomic profiling of tumour tissue or plasma samples before systemic treatment was performed using multiple targeted next‐generation sequencing (NGS) panels, according to the protocol approved by the Ethics Committee of Guangdong Provincial People's Hospital. Tumour genomic DNA was extracted from formalin‐fixed paraffin‐embedded (FFPE) samples using the QIAamp DNA FFPE Tissue Kit (QIAGEN, Dusseldorf, Germany). Genomic DNA was extracted from leukocyte (normal blood controls) using the QIAamp Circulating Nucleic Acid Kit (QIAGEN). Peripheral blood was collected and centrifuged (at 1800 ***g*** for 10 min at room temperature) within 2 h to separate the plasma and leukocytes. Cell‐free DNA was extracted from the plasma using a QIAamp Circulating Nucleic Acid Kit (QIAGEN). Sequencing libraries were prepared using the KAPA Hyper Prep Kit (KAPA Biosystems, Wilmington, MA, USA). Briefly, fragmented genomic DNA was subjected to end‐repair, A‐tailing, adapter ligation, size selection, polymerase chain reaction amplification, and purification sequentially. Target enrichment was performed using a customized xGen Lockdown Probes Panel (Integrated DNA Technologies, Coralville, IA, USA), human cot‐1 DNA (Life Technologies) and xGen Universal Blocking Oligos (Integrated DNA Technologies). All procedures were performed according to the manufacturer's instructions. The enriched libraries were sequenced using the Illumina Hiseq4000 NGS platforms (Illumina, San Diego, CA, USA).

trimmomatic was used for quality control of fastq files by removing leading/trailing low quality (reading < 15) or N bases [30]. Sequencing data were then aligned to the reference human genome (build hg19) and processed using the picard suite and the genome analysis toolkit (gatk) [31, 32]. A somatic mutation, filtered for common single nucleotide polymorphisms and germline mutations, was retained when it had at least 1% mutant allele frequency and at least three unique reads on different strands with good quality scores. Gene fusions and copy number variations were analysed using FACTERA and ADTEx [33, 34], respectively, and manually reviewed using integrative genomics viewer Software (igv; Broad Institute, Cambridge, MA, USA). A total of 72 overlapping cancer‐relevant genes from multiple NGS panels were included in the data analysis (Table S1).

### 2.4 Immunohistochemistry (IHC) for PD‐L1 expression

Formalin‐fixed paraffin‐embedded tumour tissue specimens were stained using PD‐L1 IHC 22C3 pharmDx (Agilent, Santa Clara, CA, USA), and a PD‐L1‐positive cell was defined as complete circumferential or partial cell membrane staining of viable cells with 1+ to 3+ intensity. PD‐L1 protein expression was evaluated using the tumour proportion score (TPS), which was calculated as the percentage of PD‐L1‐positive tumour cells divided by total tumour cells. Patients were categorized into three subgroups according to PD‐L1 TPS: < 1%, 1–49%, and ≥ 50%.

### 2.5 Statistical analysis

Progression‐free survival was defined as the time from the initiation of ICI‐based therapy to disease progression or death from any cause; overall survival (OS) was defined as the time from the initiation of ICI‐based therapy to death from any cause. The median follow‐up time for the GLCI‐ICI cohort was estimated using the observation time method. Fisher's exact test and two‐sample *t*‐test were performed to compare the frequencies and means of patients with *HER2* ex20ins and *HER2* non‐ex20ins mutations, respectively. For survival data, Kaplan–Meier curves for PFS and OS were generated, and log‐rank tests were used to compare differences. Hazard ratios (HR) with 95% confidence intervals (CI) were estimated using Cox proportional hazards models. Multivariable Cox proportional hazards models included clinical and molecular features that were identified as having a potentially strong influence on PFS or OS in univariate analyses or with significantly unbalanced distribution between *HER2* ex20ins and non‐ex20ins patients. The proportionality of hazards was assessed using log(−log) survival plots. Individuals with missing data were excluded from analysis. All quoted *P*‐values were two‐tailed, and *P*‐values < 0.05 were considered to be statistically significant. Data were analysed using r software (version 4.0.3, Vienna, Austria) and the *survival* package.

## 3 Results

### 3.1 Patient characteristics

A total of 107 eligible patients (76 with *HER2* ex20ins and 31 with *HER2* non‐ex20ins mutations) were retrospectively enrolled in the GLCI cohort, with only one patient identified to have ex20ins and non‐ex20ins mutations simultaneously. Thirty‐five of patients were identified in the GLCI‐ICI cohort, all of whom received ICI‐based therapy (Fig. 1). The median age of the GLCI cohort was 59 years (range: 24–81). It presented as a majority of adenocarcinoma (99/107, 92.5%), males (60/107, 56.1%), stage IV at initial diagnosis (73/107, 68.2%), and never‐smokers (78/107, 72.9%) (Table 1). Most patients (73/107, 68.2%) had PD‐L1 TPS of < 1%. Compared with patients with *HER2* ex20ins mutations, non‐ex20ins patients were older (63 *vs*. 57 years, *P* < 0.01), had a greater proportion of smokers (41.9% *vs*. 21.1%, *P* = 0.03), and had a lower proportion of adenocarcinoma (83.9% *vs*. 96.1%, *P* = 0.02). Two subgroups had similar PD‐L1 expression levels, with over 65% of patients identified with PD‐L1 expression < 1% (< 1%: 71.0% *vs*. 67.1%, 1–49%: 22.6 *vs*. 26.3, ≥ 50%: 6.5% *vs*. 6.6%, *P* = 0.94).

**Table 1:** Demographics and clinical characteristics of GLCI cohort.

Characteristics	Overall (*n* = 107)	Ex20ins (*n* = 76)	Non‐ex20ins (*n* = 31)	*P*‐value
Age, median (range), year	59 (24–81)	57 (24–79)	63 (43–81)	< 0.01*
Age, no. (%)				
< 60 years	58 (54.2)	47 (61.8)	11 (35.5)	0.01*
≥ 60 years	49 (45.8)	29 (38.2)	20 (64.5)	
Sex, no. (%)				
Female	47 (43.9)	35 (46.1)	13 (41.9)	0.83
Male	60 (56.1)	41 (53.9)	18 (58.1)	
Clinical stage at initial diagnosis, no. (%)				
I	10 (9.3)	7 (9.2)	3 (9.7)	0.73
II	6 (5.6)	5 (6.6)	1 (3.2)	
III	18 (16.8)	11 (14.5)	7 (22.6)	
IV	73 (68.2)	53 (69.7)	20 (64.5)	
Histology, no. (%)				
Adenocarcinoma	99 (92.5)	73 (96.1)	26 (83.9)	0.02*
Squamous cell carcinoma	5 (4.9)	1 (1.3)	4 (12.9)	
Adenosquamous carcinoma	1 (0.9)	1 (1.3)	0 (0.0)	
Lymphoepithelioma‐like carcinoma	1 (0.9)	0 (0.0)	1 (3.2)	
Not otherwise specified	1 (0.9)	1 (1.3)	0 (0.0)	
Smoking, no. (%)				
Ever	29 (27.1)	16 (21.1)	13 (41.9)	0.03*
Never	78 (72.9)	60 (78.9)	18 (58.1)	
PD‐L1 expression, no. (%)				
< 1%	73 (68.2)	51 (67.1)	22 (71.0)	0.94
1–49%	27 (25.2)	20 (26.3)	7 (22.6)	
≥ 50%	7 (6.5)	5 (6.6)	2 (6.5)	

* Statistically significant.

### 3.2 Genomic features and molecular heterogeneity

**Fig. 2:**
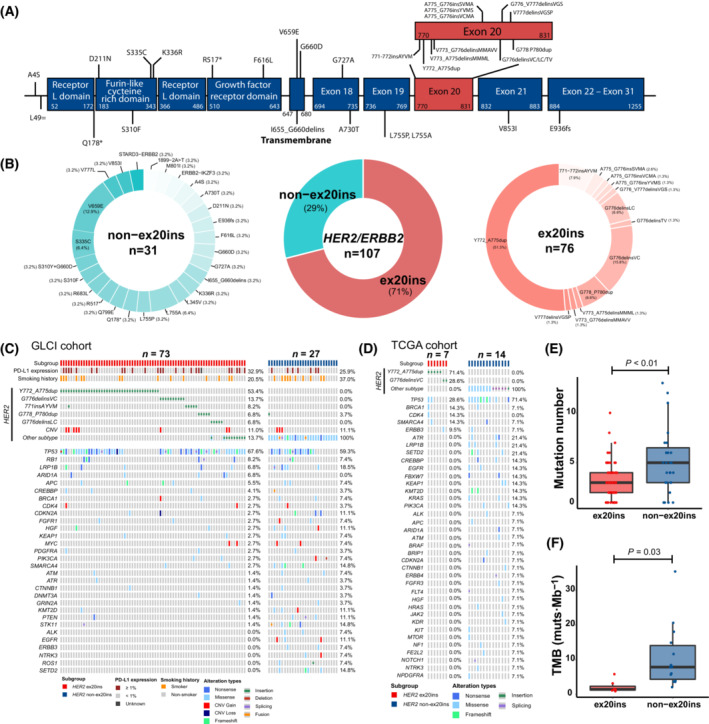
Genomic and immune features in NSCLC patients harbouring *HER2* ex20ins and non‐ex20ins mutations. (A) The lollipop plot of *HER2* ex20ins and non‐ex20ins mutations in the study cohort. (B) The category and proportion of *HER2* ex20ins and non‐ex20ins mutations. (C) Tumour tissue/plasma samples were performed using multiple targeted NGS panels, and 72 overlapping NSCLC relevant genes carried by at least three patients were presented. (D) The genomic profile of The Cancer Genome Atlas Program (TCGA) cohort, including 21 NSCLC patients carrying *HER2* mutations. (E) Mutation numbers of NSCLC patients with *HER2* ex20ins and *HER2* non‐ex20ins mutations. Error bars: interquartile range. *P*‐value: two‐sample *t*‐test. (F) TMB of NSCLC patients with *HER2* ex20ins and *HER2* non‐ex20ins mutations. Error bars: interquartile range. *P*‐value: two‐sample *t*‐test.

Of the 107 patients in the GLCI cohort, seven patients had only *HER2* alteration records before systemic treatment, without raw sequencing data. The remaining 100 patients (73 with *HER2* ex20ins and 27 with *HER2* non‐ex20ins mutations) were included in the molecular feature analyses, and their *HER2* mutations are summarized in Fig. 2A. The most frequently identified *HER2* ex20ins subtype was Y772_A775dup (39/100, 39.0%), followed by G776delinsVC (10/100, 10.0%), 771insAYVM (6/100, 6.0%), G778_P780dup (5/100, 5.0%), and G776delinsLC (5/100, 5.0%) (Fig. 2B). *HER2* non‐ex20ins mutations included V659E, L755A, S335C, etc. (Fig. 2B). The most commonly mutated passenger gene was *TP53* (ex20ins, 67.6%; non‐ex20ins, 59.3%), followed by *LRP1B* (ex20ins, 6.8%; non‐ex20ins, 18.5%) and *RB1* (ex20ins, 8.2%; non‐ex20ins, 7.4%) (Fig. 2C).

Compared with patients with *HER2* ex20ins mutations, altered *SETD2* gene (0.0% *vs*. 14.8%, *P* < 0.01) was more frequently observed in non‐ex20ins patients in the GLCI‐ICI cohort (Fig. 2C). In the TCGA cohort [seven with *HER2* ex20ins and 14 with *HER2* non‐ex20ins mutations, median age: 67 years (range: 52–81), females 66.7%, Table S2], despite a lower *TP53* alteration prevalence in *HER2* ex20ins patients (28.6%), similar frequently altered genes were observed in comparison with the GLCI cohort, such as *BRCA1*, *CDK4*, and *LRP1B* (Fig. 2D). In the GLCI cohort, the average number of mutations identified in one biopsy was 3.7, and patients with *HER2* non‐ex20ins mutations had more concurrent mutations than ex20ins patients (5.2 *vs*. 2.1, *P* < 0.01, Fig. 2E). Similarly, in the TCGA cohort, non‐ex20ins patients had higher TMB than ex20ins patients (10.7 *vs*. 2.2 muts·Mb^−1^, *P* = 0.03, Fig. 2F).

### 3.3 Immune microenvironment features

To gain more insight into the TME of patients with *HER2* mutations, transcriptome data from the TCGA cohort were analysed using the CIBERSORT algorithm, and the degree of immune cell infiltration was estimated. Among the 21 patients in the TCGA cohort, *HER2* non‐ex20ins patients might have a potentially higher density of resting CD4^+^ memory T cells than *HER2* ex20ins patients, while the difference was not statistically significant (15.6% *vs*. 12.3%, *P* = 0.54, Fig. S1A).

### 3.4 Clinical efficacy of ICI‐based therapy

The genomic and TME features suggested that the efficacy of ICIs in advanced NSCLC patients with *HER2* mutations was possibly associated with the subtype of *HER2* mutation. Next, we analysed the efficacy of ICIs in the GLCI‐ICI cohort (23 with *HER2* ex20ins, 12 with *HER2* non‐ex20ins mutations, and one with missing PFS data). The median follow‐up time was 11.6 (range: 0.3–70.0) months. Patients with PD‐L1 TPS < 1% were frequently observed (22/35, 62.9%), and most of the patients received ICI therapy as the second‐line or subsequent treatment (28/35, 80.0%) (Table 2). Of 35 patients in the GLCI‐ICI cohort, 31 had available TMB data, and the percentage of patients with high TMB (≥ 10 muts·Mb^−1^) appeared to be higher in the non‐ex20ins subgroup than in the ex20ins subgroup (41.7% *vs*. 21.7%, *P* = 0.24). The other clinical characteristics (Table 2) and genomic profiles of the GLCI‐ICI cohort were similar to those of the entire study cohort (Fig. S1B).

**Table 2:** Demographics and clinical characteristics of GLCI‐ICI cohort.

Characteristics	Overall (*n* = 35)	Ex20ins (*n* = 23)	Non‐ex20ins (*n* = 12)	*P*‐value
Age, median (range), year	56 (35–72)	54 (35–72)	58 (51–72)	0.11
Age, no. (%)				
< 60 years	23 (65.7)	16 (69.6)	7 (58.3)	0.71
≥ 60 years	12 (34.3)	7 (30.4)	5 (41.7)	
Sex, no. (%)				
Female	12 (34.3)	8 (34.8)	4 (33.3)	> 0.99
Male	23 (65.7)	15 (65.2)	8 (66.7)	
Clinical stage at initial diagnosis, no. (%)				
IIIb	2 (5.7)	0 (0.0)	2 (16.7)	0.20
IVa	15 (42.9)	10 (43.5)	5 (41.7)	
IVb	18 (51.4)	13 (56.5)	5 (41.7)	
Histology, no. (%)				
Adenocarcinoma	32 (91.4)	22 (95.7)	10 (83.3)	0.27
Squamous cell carcinoma	3 (8.6)	1 (4.3)	2 (16.7)	
Smoking, no. (%)				
Ever	13 (37.1)	7 (30.4)	6 (50.0)	0.30
Never	22 (62.9)	16 (69.6)	6 (50.0)	
ECOG performance status, no. (%)				
1	33 (94.3)	21 (91.3)	12 (100.0)	> 0.99
2	1 (2.9)	1 (4.3)	0 (0.0)	
3	1 (2.9)	1 (4.3)	0 (0.0)	
PD‐L1 expression, no. (%)				
< 1%	22 (62.9)	12 (52.2)	9 (75.0)	0.65
1–49%	12 (34.3)	9 (39.1)	3 (25.0)	
≥ 50%	1 (3.9)	1 (4.3)	0 (0.0)	
TMB, no. (%)				
< 10 muts·Mb^−1^	19 (54.3)	15 (65.2)	4 (33.3)	0.24
≥ 10 muts·Mb^−1^	10 (28.6)	5 (21.7)	5 (41.7)	
Unknown	6 (17.1)	3 (13.0)	3 (25.0)	
ICI‐based therapy, no. (%)				
Monotherapy	15 (42.9)	8 (34.8)	7 (58.3)	0.28
Combination therapy	20 (57.1)	15 (65.2)	5 (41.7)	
ICI lines, no. (%)				
1st	7 (20.0)	3 (13.0)	4 (33.3)	0.20
≥ 2nd	28 (80.0)	20 (87.0)	8 (66.7)	

**Fig. 3:**
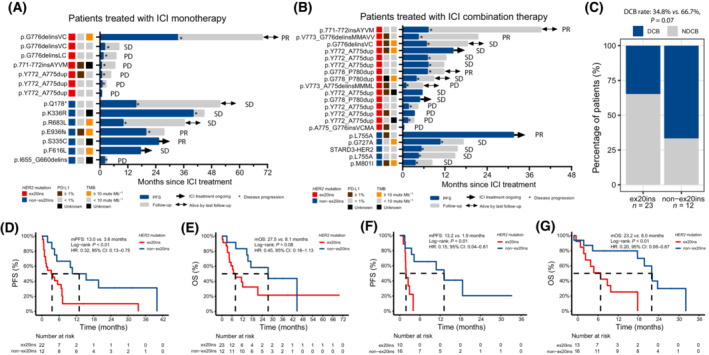
Swimmer plots, best of response, and survival of *HER2*‐mutated NSCLC patients receiving ICI‐based therapy. (A) A total of 15 *HER2*‐mutated NSCLC patients were treated with ICI monotherapy, including seven harbouring *HER2* ex20ins and eight harbouring *HER2* non‐ex20ins mutations (one patient with missing PFS data was not presented here). (B) Other 20 *HER2*‐mutated NSCLC patients were treated with ICI combination therapy, including 15 ex20ins patients and five patients harbouring *HER2* non‐ex20ins mutations. (C) Patients with *HER*2 non‐ex20ins mutations had a relatively high durable clinical benefit (complete/partial response or stable disease lasting over 6 months) rate in comparison with patients harbouring *HER2* ex20ins (66.7% *vs*. 34.8%, *P* = 0.07, Fisher's exact test). (D, E) In the GLCI‐ICI cohort, *HER2*‐mutated NSCLC patients with *HER2* non‐ex20ins displayed potentially better PFS and OS than those with *HER2* ex20ins mutations. (F, G) In the META‐ICI cohort, similar results were obtained that non‐ex20ins patients had significantly superior PFS and OS than ex20ins patients.

Fifteen patients in the GLCI‐ICI cohort were treated with ICI monotherapy (Fig. 3A), and the remaining 20 patients received ICI combination therapy (Fig. 3B). The overall ORR was 20.0% (95% CI: 8.0–41.2%); the mPFS and median OS (mOS) of the GLCI‐ICI cohort were 5.2 (95% CI: 3.5–9.9) months and 14.8 (95% CI: 8.1–not reached) months, respectively. Patients carrying *HER2* non‐ex20ins appeared to achieve relatively high disease control rates compared with those carrying *HER2* ex20ins mutations (monotherapy, 85.7% *vs*. 28.6%, *P* = 0.10; ICI combination therapy, 100% *vs*. 66.7%, *P* = 0.27). Also, patients with *HER2* non‐ex20ins mutations exhibited a relatively high durable clinical benefit (complete/partial response or stable disease lasting over 6 months) compared with those with *HER2* ex20ins (66.7% *vs*. 34.8%, *P* = 0.07, Fig. 3C); however, they did not have a significantly higher ORR (25.0% *vs*. 17.4%, *P* = 0.67). Details of treatment and ICI efficacy in the GLCI‐ICI cohort are shown in Table 3.

**Table 3:** Treatment plan and efficacy of GLCI‐ICI cohort.

Patient ID	*HER2* mutation	Subtype	Sex	Age over 60	Smoking	Adenocarcinoma	TMB over 10 muts·Mb^−1^	PD‐L1 over 1%	Clinical stage	ICI line	ICI treatment plan	Clinical response	PFS status	PFS (months)	OS status	OS (months)
HER2‐106	Exon 20ins	p.Y772_A775dup	Female	Yes	Never	Yes	No	Yes	IVA	3rd	Pembrolizumab	PD	1	0.7	1	4.57
HER2‐111	Non‐exon 20ins	p.Q178*	Male	No	Ever	No	No	No	IVA	5th	Tislelizumab (BGB‐A317)	SD	1	15.4	0	22.77
HER2‐125	Non‐exon 20ins	p.L755A	Female	No	Never	Yes	No	No	IVA	1st	Pemetrexed + Carboplatin + Pembrolizumab	SD	1	4.4	1	14.83
HER2‐136	Non‐exon 20ins	STARD3‐ERBB2	Female	No	Never	Yes	No	No	IVA	2nd	Pembrolizumab + Docetaxel	SD	1	5.2	1	15.50
HER2‐144	Exon 20ins	p.G776delinsVC	Male	No	Never	Yes	Yes	Yes	IVB	2nd	Pemetrexed + Carboplatin + Pembrolizumab	SD	1	6.7	0	18.33
HER2‐148	Non‐exon 20ins	p.L755A	Female	Yes	Never	Yes	No	Yes	IVA	1st	Nab‐Pacilitaxel + Carboplatin + Pembrolizumab	PR	0	31.5	0	31.50
HER2‐159	Exon 20ins	p.Y772_A775dup	Female	No	Never	Yes	No	No	IVA	3rd	Camrelizumab + Bevacizumab	SD	1	7.0	1	12.37
HER2‐164	Non‐exon 20ins	p.K336R	Female	Yes	Never	Yes	NA	No	IVA	2nd	Camrelizumab	SD	1	40.0	1	44.73
HER2‐18	Non‐exon 20ins	p.R683L	Male	Yes	Ever	Yes	Yes	No	IVB	1st	Nivolumab	SD	1	9.9	0	36.30
HER2‐181	Exon 20ins	p.Y772_A775dup	Male	Yes	Never	Yes	No	No	IVA	3rd	Pemetrexed + Carboplatin + Pembrolizumab	SD	1	7.2	1	11.70
HER2‐187	Non‐exon 20ins	p.M801I	Male	No	Ever	No	Yes	Yes	IIIB	2nd	Docetaxel + Carboplatin + Pembrolizumab	SD	1	3.5	1	8.30
HER2‐194	Exon 20ins	p.V773_G776delinsMMAVV	Male	No	Ever	Yes	No	Yes	IVB	1st	Pemetrexed + Carboplatin + Pembrolizumab	PR	1	4.4	1	21.40
HER2‐2	Exon 20ins	p.Y772_A775dup	Male	No	Never	Yes	No	No	IVB	3rd	Pembrolizumab	PD	1	0.9	1	2.60
HER2‐200	Exon 20ins	p.Y772_A775dup	Male	Yes	Ever	Yes	NA	Yes	IVA	2nd	Pemetrexed + Carboplatin + Pembrolizumab	SD	1	5.5	1	5.47
HER2‐202	Exon 20ins	p.Y772_A775dup	Female	Yes	Never	Yes	Yes	Yes	IVB	2nd	Pemetrexed + Bevacizumab + Sintilimab	SD	0	14.3	0	14.33
HER2‐241	Non‐exon 20ins	p.F616L	Male	No	Ever	Yes	Yes	No	IVB	2nd	Nivolumab	SD	0	17.4	0	17.40
HER2‐244	Exon 20ins	p.G778_P780dup	Male	No	Never	Yes	Yes	NA	IVA	2nd	Nab‐Pacilitaxel + Carboplatin + Pembrolizumab	SD	1	3.8	0	8.80
HER2‐264	Exon 20ins	p.Y772_A775dup	Female	No	Never	Yes	No	Yes	IVB	2nd	Pemetrexed + Carboplatin + Camrelizumab	PD	1	3.3	1	3.33
HER2‐280	Exon 20ins	p.G778_P780dup	Male	No	Ever	Yes	No	No	IVA	2nd	Pacilitaxel + Pembrolizumab	PR	1	7.0	0	11.63
HER2‐283	Exon 20ins	p.G778_P780dup	Male	Yes	Never	Yes	No	No	IVA	3rd	Pemetrexed + Sintilimab	SD	0	4.8	0	4.77
HER2‐286	Exon 20ins	p.A775_G776insVCMA	Male	No	Ever	Yes	No	Yes	IVB	2nd	Pemetrexed + Carboplatin + ICI (unknown)	PD	1	0.3	1	0.27
HER2‐287	Exon 20ins	p.V773_A775delinsMMML	Male	No	Never	Yes	No	Yes	IVB	2nd	Pemetrexed + Carboplatin + Pembrolizumab	PD	1	0.9	0	5.73
HER2‐32	Non‐exon 20ins	p.I655_G660delins	Male	No	Never	Yes	NA	No	IVB	5th	Nivolumab	PD	1	2.0	1	2.97
HER2‐33	Non‐exon 20ins	p.E936fs	Male	Yes	Ever	Yes	Yes	Yes	IVB	3rd	Pembrolizumab	PR	1	19.7	1	27.53
HER2‐35	Non‐exon 20ins	p.G727A	Male	Yes	Ever	Yes	Yes	No	IIIB	1st	Pacilitaxel + Carboplatin + Durvalumab	SD	1	10.6	1	17.27
HER2‐4	Exon 20ins	p.Y772_A775dup	Male	Yes	Ever	Yes	No	No	IVB	3rd	Nivolumab	PD	1	0.7	1	0.70
HER2‐41	Exon 20ins	p.G776delinsVC	Male	No	Ever	Yes	No	No	IVB	4th	Pembrolizumab	SD	1	2.0	1	8.07
HER2‐55	Exon 20ins	p.Y772_A775dup	Female	No	Never	Yes	NA	No	IVB	4th	Nivolumab + Bevacizumab	PD	1	1.4	1	3.27
HER2‐60	Non‐exon 20ins	p.S335C	Male	No	Never	Yes	NA	No	IVB	3rd	Nivolumab	PR	0	25.2	0	25.17
HER2‐62	Exon 20ins	p.G776delinsVC	Female	No	Never	Yes	Yes	No	IVA	3rd	Nivolumab	PR	1	33.5	0	70.00
HER2‐68	Exon 20ins	p.Y772_A775dup	Male	Yes	Ever	Yes	No	No	IVB	1st	Pemetrexed + Carboplatin + Pembrolizumab	PD	1	1.6	1	4.30
HER2‐72	Exon 20ins	p.771‐772insAYVM	Male	No	Never	No	No	No	IVA	3rd	Pacilitaxel + Pembrolizumab	PR	1	7.2	0	39.27
HER2‐74	Exon 20ins	p.G776delinsLC	Female	No	Never	Yes	No	No	IVA	4th	KL‐A167	PD	1	1.3	1	6.90
HER2‐82	Exon 20ins	p.771‐772insAYVM	Male	No	Never	Yes	NA	Yes	IVB	1st	Pembrolizumab	PD	1	0.6	1	6.43
HER2‐83	Exon 20ins	p.771‐772insAYVM	Female	No	Never	Yes	Yes	Yes	IVB	4th	Nivolumab	–	–	–	1	4.37

After excluding one patient with unavailable PFS data, 12 patients with *HER2* non‐ex20ins mutations had an mPFS of 13.0 months, and they might have superior PFS to ex20ins patients (HR: 0.32, 95% CI: 0.13–0.75, Fig. 3D). Non‐ex20ins patients might also have longer mOS (27.5 *vs*. 8.1 months) and relatively good OS in comparison with patients carrying *HER2* ex20ins (HR: 0.45, 95% CI: 0.18–1.13, Fig. 3E). The influence of potential confounders on prognosis was also investigated (Table 4). Patients whose TMB ≥ 10 muts·Mb^−1^ had a relatively low risk of disease progression than those with TMB < 10 muts·Mb^−1^ (HR: 0.40, 95% CI: 0.16–1.00), and a potential association of TMB with OS was also observed (HR: 0.35, 95% CI: 0.11–1.09). None of the patients' age, sex, histologic type, smoking history, PD‐L1 expression, ICI‐based therapy regimen, and whether ICI‐based therapy served as first‐line treatment, showed a strong association with PFS or OS. In the multivariable Cox regression model controlling for age, histologic type, smoking history, and TMB, *HER2* non‐ex20ins mutations remained associated with potentially better PFS (adjusted HR: 0.31, 95% CI: 0.11–0.83) and OS (adjusted HR: 0.39, 95% CI: 0.13–1.18).

**Table 4:** Hazard ratios estimated by univariate and multivariable Cox regression models in GLCI‐ICI cohort. Ref, reference.

Characteristics	No. of patients (%)	PFS				OS			
		Univariate		Multivariable		Univariate		Multivariable	
		HR (95% CI)	*P*	HR (95% CI)	*P*	HR (95% CI)	*P*	HR (95% CI)	*P*
*ERBB2* mutation									
Ex20ins	12 (34.3)	Ref		Ref		Ref		Ref	
Non‐ex20ins	23 (65.7)	0.32 (0.13–0.75)	< 0.01*	0.31 (0.11–0.83)	0.02*	0.45 (0.18–1.13)	0.09	0.39 (0.13–1.18)	0.10
Age									
< 60 years	23 (65.7)	Ref		Ref		Ref		Ref	
≥ 60 years	12 (34.3)	0.49 (0.21–1.12)	0.09	0.55 (0.23–1.32) 0.18		0.93 (0.38–2.23)	0.87	1.06 (0.40–2.83) 0.90	
Sex									
Female	12 (34.3)	Ref		–^a^		Ref		–^a^	
Male	23 (65.7)	1.60 (0.67–3.80)	0.29	–^a^		0.91 (0.38–2.20)	0.83	–^a^	
Histology									
Squamous cell carcinoma	32 (91.4)	Ref		Ref		Ref		Ref	
Adenocarcinoma	3 (8.6)	1.02 (0.31–3.40)	0.98	1.61 (0.41–6.26) 0.49		2.91 (0.39–21.78)	0.30	3.60 (0.44–29.28) 0.23	
Smoking									
Ever	13 (37.1)	Ref		Ref		Ref		Ref	
Never	22 (62.9)	0.83 (0.39–1.78)	0.63	0.54 (0.24–1.18) 0.12		0.85 (0.36–2.01)	0.70	0.61 (0.25–1.50) 0.28	
PD‐L1 expression									
< 1%	22 (62.9)	Ref		–^a^		Ref		–^a^	
≥ 1%	12 (34.3)	1.41 (0.64–3.313)	0.39	–^a^		1.41 (0.59–3.36)	0.43	–^a^	
Unknown	1 (3.9)	2.62 (0.29–17.77)	0.44	–^a^		–^b^		–^a^	
TMB									
< 10 muts·Mb^−1^	19 (54.3)	Ref		Ref		Ref		Ref	
≥ 10 muts·Mb^−1^	10 (28.6)	0.40 (0.16–1.00)	0.05	0.39 (0.14–1.09)	0.07	0.35 (0.11–1.09)	0.07	0.32 (0.10–1.05)	0.06
Unknown	6 (17.1)	0.47 (0.15–1.47)	0.20	0.82 (0.24–2.86)	0.76	1.04 (0.35–3.07)	0.94	1.37 (0.41–4.53)	0.61
ICI‐based treatment									
Monotherapy	15 (42.9)	Ref		–^a^		Ref		–^a^	
Immunochemotherapy	20 (57.1)	1.49 (0.65–3.40)	0.34	–^a^		1.11 (0.90–2.67)	0.82	–^a^	
ICI line									
1st	7 (20.0)	Ref		–^a^		Ref		–^a^	
≥ 2nd	28 (80.0)	0.96 (0.39–2.39)	0.93	–^a^		1.13 (0.41–3.09)	0.82	–^a^	

^a^ Not included in multivariable Cox regression models.

^b^ Insufficient patient number for model fitting.

* Statistically significant.

To validate the findings in the GLCI‐ICI cohort, 30 eligible patients from external data sets of ICI‐based therapy studies were grouped into the META‐ICI cohort (13 with *HER2* ex20ins and 17 with *HER2* non‐ex20ins mutations, four with unavailable PFS data, and one with unavailable OS data, Table S3). Notably, the majority of patients in the META‐ICI cohort were treated with ICI monotherapy. The mPFS and mOS of the META‐ICI cohort were 4.0 (95% CI: 1.9–not reached) months and 18.9 (95% CI: 8.0–24.9) months, respectively. The demographic and clinical characteristics of the META‐ICI cohort are summarized in Table S3. The mPFS and mOS of patients harbouring *HER2* ex20ins were 1.9 and 8.0 months, respectively, and patients with *HER2* non‐ex20ins mutations presented a longer mPFS of 13.2 months and a longer mOS of 23.3 months. *HER2* non‐ex20ins mutations were associated with favourable PFS (HR: 0.15, 95% CI: 0.04–0.61, Fig. 3F) and OS (HR: 0.20, 95% CI: 0.06–0.67, Fig. 3G).

## 4 Discussion

In this study, we analysed the molecular and TME characteristics of NSCLC patients with *de novo HER2* mutations, as well as their responses to immunotherapy. *HER2* non‐ex20ins patients had higher mutation numbers than *HER2* ex20ins patients, whereas similar low‐level PD‐L1 expression was detected. *HER2* non‐ex20ins mutations were potentially associated with superior PFS and OS under ICI‐based therapy, which was consistent with the findings in external cohorts.

Based on the GLCI cohort, we provided a landscape view of the genomic and clinical features of 107 *HER2*‐mutated NSCLC patients without common driver mutations. The diversity of *HER2* mutations suggested potentially uniform responses to the same treatment. In this study, *HER2* ex20ins mutations were detected in 76 (71.0%) patients, similar to the results of another East Asian cohort study by Tan et al. (72.7%) [35]. In contrast, a study based on a western cohort (*n* = 84) revealed that *HER2* ex20ins accounted for 34.4% *HER2* mutations [6]. For concurrent *TP53* mutations, the prevalence was similar between patients harbouring *HER2* ex20ins and non‐ex20ins mutations in our study (67.6% *vs*. 59.3%), whereas the prevalence in the TCGA cohort appeared to be relatively high in the non‐ex20ins subgroup (28.6% *vs*. 71.4%). Consistent with the TMB results reported by Tan et al. [35], *HER2* non‐ex20ins patients in our study had higher mutation numbers than patients with *HER2* ex20ins mutations.

Our data provide preliminary insights into the TME heterogeneity in *HER2*‐mutated NSCLC. Similar to the results of a previous study [36], a generally immunosuppressed TME was observed, whereas a heterogeneous TME was observed in *HER2*‐mutated NSCLC. In our study, patients with *HER2* ex20ins and non‐ex20ins mutations showed similar PD‐L1 TPS, with over 60% of the patients having negative expression levels (< 1%), which aligned with the findings of previous studies [5, 20, 35]. In the TCGA cohort, non‐ex20ins patients might be enriched with resting CD4^+^ memory T cells, which should be further investigated in a large cohort of *HER2*‐mutated NSCLC patients.

Our findings suggest that ICI‐based therapy might serve as a treatment option for advanced NSCLC patients with *HER2* mutations, and that those harbouring *HER2* non‐ex20ins mutations could potentially benefit more. In the GLCI‐ICI cohort, the mPFS and ORR of ICI‐based therapy were 5.2 months and 20.0%, respectively, consistent with the result of previous studies [20, 21, 22, 23]. In the German nNGM lung cancer cohort, a 26.2% ORR was reported in 61 *HER2*‐mutated NSCLC patients receiving ICI‐based therapy as the first‐line and later lines of treatment [20]. In the French Lung Cancer Group 01‐2018 and MSKCC research, ICI‐based therapy showed ORRs of 27.3% and 11.5%, respectively [21, 22]. In our study, a potentially better response to ICI was observed in patients harbouring *HER2* non‐ex20ins mutations than in those harbouring *HER2* ex20ins, which was similar to the findings of previous studies [5, 37, 38, 39, 40]. For instance, as reported in Fan's study, none of the six *HER2* ex20ins patients treated with PD‐1 inhibitors achieved an objective response [38]. In a study by Tan et al. [35], none of the four ex20ins patients achieved a response after receiving pembrolizumab monotherapy. Our study also revealed that non‐ex20ins patients were more likely to achieve a better response to ICI‐based therapy than ex20ins patients; however, further research with a larger sample size is warranted. The relatively poor prognosis of ex20ins patients could be partially explained by the low TMB levels in patients harbouring *HER2* ex20ins [29, 41], as advanced NSCLC patients with higher TMB (above 50% percentile) could achieve better PFS than patients with lower TMB (below 50% percentile) [25]. Mutated *SETD2*, enriched in non‐ex20ins NSCLC, was identified as a favourable predictive biomarker for immunotherapy, associated with higher TMBs and better OS (HR: 0.55, 95% CI: 0.46–0.65) [42]. *LRP1B* mutations, being relatively frequent in *HER2* non‐ex20ins patients, were also associated with prolonged survival (HR: 0.63, 95% CI: 0.40–0.97) [43]. In our study, we did not detect a strong association between PD‐L1 expression and PFS/OS, which might have resulted from the similar proportions of patients with at least 1% PD‐L1 expression between the ex20ins and non‐ex20ins subgroups.

In the era of *HER2*‐ADC, comprehensive studies including considerable *HER2* non‐ex20ins patients were warranted to investigate how *HER2* mutation subtype affects ADC efficacy and whether immunotherapy or *HER2*‐ADC would be the optimal first‐line regimen for NSCLC patients with specific subtypes of *HER2* mutations. In DESTINY‐Lung01, *HER2* non‐ex20ins patients did not achieve as good response to T‐DXd as ex20ins patients (ORR: 33% *vs*. 73%, *P* = 0.02) [15], suggesting that the efficacy of T‐DXd might depend on *HER2* mutation subtype. For *HER2*‐mutated patients in East Asia, Tan et al. reported that three of four (75.0%) ex20ins patients responded to T‐DXd; however, the efficacy of ADCs in non‐ex20ins patients remained unclear. In our study, *HER2* non‐ex20ins patients achieved ORR of 25.0% and mPFS of 13.0 months. Further studies comparing the treatment efficacy of *HER2*‐ADC and immunotherapy might be useful for the treatment of advanced NSCLC patients with *HER2* non‐ex20ins mutations.

Our study has some limitations. As this was a retrospective study, the timing of the disease progression assessment was not standardized. The potentially superior PFS in non‐ex20ins patients might be influenced by the line of therapy, which could not be well controlled, owing to the limited sample size of the GLCI‐ICI cohort. In addition, multiple NGS panels were performed on participants' tumour tissue or plasma samples, and only overlapping cancer‐relevant genes were included for data analyses, resulting in the failure to accurately calculate TMB in the GLCI cohort and compare key ICI treatment‐related signalling pathways. We presented the number of mutations per person instead of the TMB value in the GLCI cohort, even though TMB data were available for patients in the GLCI‐ICI cohort. Another limitation is the limited sample size of the GLCI‐ICI cohort, resulting in an inability to comprehensively analyse the prognostic data for patients treated with ICI monotherapy or combination therapy, separately. Additionally, the results of the TME comparison were not comprehensive, owing to the lack of RNA‐Seq data in the GLCI‐ICI cohort.

## 5 Conclusion

Our study revealed that *HER2*‐mutated lung cancers present with a high‐level molecular heterogeneity. Patients harbouring *HER2* non‐ex20ins mutations had higher mutation numbers than patients harbouring *HER2* ex20ins mutations, whereas similar PD‐L1 expression levels were detected. *HER2* non‐ex20ins mutations could potentially be considered positive predictors of ICI efficacy in advanced *HER2*‐mutated NSCLC patients, and ICI‐based therapy might be a good option for patients with *HER2* non‐ex20ins mutations.

## Conflict of interest

Yi‐Long Wu has received honoraria from AstraZeneca, Eli Lilly, Roche, Pierre Fabre, Pfizer, and Sanofi; consulting or advisory role with AstraZeneca, Roche, Merck and Boehringer Ingelheim, and Roche outside the submitted work. Xiaotian Zhao, Dongqin Zhu, Lingling Yang, and Qiuxiang Ou are employees of Nanjing Geneseeq Technology Inc., China. The remaining authors have nothing to disclose.

## Author contributions

H‐YT, KY, and Y‐LW designed the study. Y‐LS, J‐XL, X‐YB, and Y‐CZ were responsible for patient recruitment and sample collection. E‐EK, S‐PW, Y‐SL, M‐MZ, S‐YML, C‐RX, QZ, J‐JY, W‐ZZ, B‐CW, and X‐CZ were responsible for patient clinical and survival data collection and results interpretation. H‐YT, KY, XZ, DZ, LY, and QO analysed data and interpreted results. All authors wrote and reviewed the manuscript, and approved the submitted version.

## Supporting information


**Fig. S1.** Transcriptomic data of The Cancer Genome Atlas Program (TCGA) cohort and the genomic profile of the Guangdong Lung Cancer Institute‐immune checkpoint inhibitor (GLCI‐ICI) cohort.

**Table S1.** 72 Overlapping cancer‐relevant genes.
**Table S2.** Demographics and clinical characteristics of TCGA cohort.
**Table S3.** Demographics and clinical characteristics of META‐ICI cohort.

## Data Availability

The data sets used and/or analysed in the current study are available from the corresponding author on reasonable request.
