# Supplementary figures and images for: Genomic and immune characteristics of HER2‐mutated non‐small‐cell lung cancer and response to immune checkpoint inhibitor‐based therapy
Source: Mol Oncol. 2023 Apr 29;17(8):1581–94. doi: 10.1002/1878-0261.13439 (PMC10399722; doi:10.1002/1878-0261.13439)

A

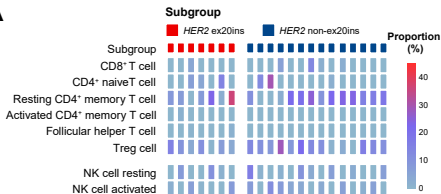

B

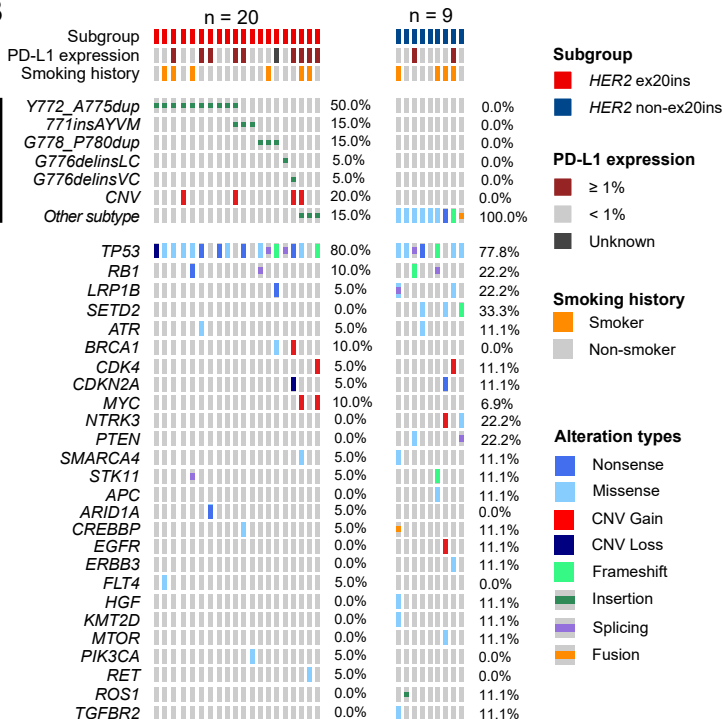

Supplement: Supplementary file 1 — Fig. S1. Transcriptomic data of The Cancer Genome Atlas Program (TCGA) cohort and the genomic profile of the Guangdong Lung Cancer Institute‐immune checkpoint inhibitor (GLCI‐ICI) cohort. [file MOL2-17-1581-s001.pdf]
