# Supplementary material for: Genomic and immune characteristics of HER2‐mutated non‐small‐cell lung cancer and response to immune checkpoint inhibitor‐based therapy
Source: Mol Oncol. 2023 Apr 29;17(8):1581–94. doi: 10.1002/1878-0261.13439 (PMC10399722; doi:10.1002/1878-0261.13439)
Supplement: Supplementary file 2 — Table S1. 72 Overlapping cancer‐relevant genes. Table S2. Demographics and clinical characteristics of TCGA cohort. Table S3. Demographics and clinical characteristics of META‐ICI cohort. [file MOL2-17-1581-s002.docx]

**Supplementary**

| **Supplementary Table 1. 72 Overlapping Cancer-relevant Genes** | | | |
| --- | --- | --- | --- |
| No. | *Gene* | No. | *Gene* |
| 1 | *AKT1* | 37 | *KEAP1* |
| 2 | *ALK* | 38 | *KIT* |
| 3 | *APC* | 39 | *KMT2D* |
| 4 | *AR* | 40 | *KRAS* |
| 5 | *ARID1A* | 41 | *LRP1B* |
| 6 | *ATM* | 42 | *MAP2K1* |
| 7 | *ATR* | 43 | *MET* |
| 8 | *BCL2L11* | 44 | *MLH1* |
| 9 | *BRAF* | 45 | *MTOR* |
| 10 | *BRCA1* | 46 | *MYC* |
| 11 | *BRIP1* | 47 | *NBN* |
| 12 | *CD274* | 48 | *NF1* |
| 13 | *CDK4* | 49 | *NFE2L2* |
| 14 | *CDK6* | 50 | *NOTCH1* |
| 15 | *CDKN1B* | 51 | *NRAS* |
| 16 | *CDKN2A* | 52 | *NTRK1* |
| 17 | *CHEK2* | 53 | *NTRK3* |
| 18 | *CREBBP* | 54 | *PDGFRA* |
| 19 | *CTNNB1* | 55 | *PDGFRB* |
| 20 | *DNMT3A* | 56 | *PIK3CA* |
| 21 | *EGFR* | 57 | *PIK3R1* |
| 22 | *ERBB2* | 58 | *PTEN* |
| 23 | *ERBB3* | 59 | *RAF1* |
| 24 | *ERBB4* | 60 | *RB1* |
| 25 | *FBXW7* | 61 | *RET* |
| 26 | *FGFR1* | 62 | *ROS1* |
| 27 | *FGFR3* | 63 | *SETD2* |
| 28 | *FLT4* | 64 | *SMAD4* |
| 29 | *GRIN2A* | 65 | *SMARCA4* |
| 30 | *HGF* | 66 | *SOX2* |
| 31 | *HRAS* | 67 | *STAG2* |
| 32 | *IDH1* | 68 | *STK11* |
| 33 | *IDH2* | 69 | *TGFBR2* |
| 34 | *JAK1* | 70 | *TP53* |
| 35 | *JAK2* | 71 | *U2AF1* |
| 36 | *KDR* | 72 | *VEGFA* |

| **Supplementary Table 2. Demographics and Clinical Characteristics of TCGA Cohort** | | | | |
| --- | --- | --- | --- | --- |
| Characteristics | Overall (n=21) | Ex20ins (n=7) | Non-ex20ins (n=14) | *P*-value |
| Age, median (range), y | 67 (52-81) | 69 (52-79) | 65 (52-81) | 0.65 |
| Age, No. (%) |  |  |  | > 0.99 |
| < 60 y | 7 (33.3) | 2 (28.6) | 5 (35.7) |  |
| ≥ 60 y | 14 (66.7) | 5 (71.4) | 9 (64.3) |  |
| Sex, No. (%) |  |  |  | > 0.99 |
| Female | 14 (66.7) | 5 (71.4) | 9 (64.3) |  |
| Male | 7 (33.3) | 2 (28.6) | 5 (35.7) |  |
| Clinical stage at initial diagnosis, No. (%) |  |  |  | 0.47 |
| I | 15 (71.4) | 4 (57.1) | 11 (78.6) |  |
| II | 3 (14.3) | 1 (14.3) | 2 (14.3) |  |
| III | 2 (9.5) | 1 (14.3) | 1 (7.1) |  |
| IV | 1 (4.8) | 1 (14.3) | 0 (0.0) |  |
| Histology, No. (%) |  |  |  | 0.05 |
| Adenocarcinoma | 7 (33.3) | 4 (57.1) | 3 (21.4) |  |
| Adenocarcinoma with mixed subtype | 5 (23.8) | 2 (28.6) | 3 (21.4) |  |
| Squamous cell carcinoma | 8 (38.1) | 0 (0.0) | 7 (50.0) |  |
| Unknown | 1 (4.8) | 1 (14.3) | 0 (0.0) |  |
| Abbreviations: Ex20ins, *HER2* mutation of exon 20 insertion; Non-ex20ins, *HER2* mutation other than exon 20 insertion | | | | |

| **Supplementary Table 3. Demographics and Clinical Characteristics of META-ICI Cohort** | | | | |
| --- | --- | --- | --- | --- |
| Characteristics | Overall (n=30) | Ex20ins (n=13) | Non-ex20ins (n=17) | *P*-value |
| Age, median (range), y | 60 (50-81) | 60 (50-66) | 60 (53-81) | 0.16 |
| Age, No. (%) |  |  |  | 0.79 |
| < 60 y | 12 (40.0) | 5 (38.5) | 7 (41.2) |  |
| ≥ 60 y | 13 (43.3) | 5 (38.5) | 8 (47.1) |  |
| Unknown | 5 (16.7) | 3 (23.1) | 2 (11.8) |  |
| Sex, No. (%) |  |  |  | 0.07 |
| Female | 17 (56.7) | 10 (76.9) | 7 (41.2) |  |
| Male | 13 (43.3) | 3 (23.1) | 10 (58.8) |  |
| Smoking, No. (%) |  |  |  | 0.02* |
| Ever | 18 (60.0) | 4 (30.8) | 14 (82.4) |  |
| Never | 8 (26.7) | 6 (46.2) | 2 (11.8) |  |
| Unknown | 4 (13.3) | 3 (23.1) | 1 (5.9) |  |
| ICI-based treatment, No. (%) |  |  |  | 0.29 |
| Monotherapy | 25 (83.3) | 10 (76.9) | 15 (88.2) |  |
| Combination therapy | 1 (3.3) | 0 (0.0) | 1 (5.9) |  |
| Unknown | 4 (13.3) | 3 (23.1) | 1 (5.9) |  |
| ICI lines, No. (%) |  |  |  | 0.05* |
| 1st | 11 (36.7) | 2 (15.4) | 9 (52.9) |  |
| ≥ 2nd | 13 (43.3) | 7 (53.8) | 6 (35.3) |  |
| Unknown | 6 (20.0) | 4 (30.8) | 1 (5.9) |  |
| Abbreviations: Ex20ins, *HER2* mutation of exon 20 insertion; Non-ex20ins, *HER2* mutation other than exon 20 insertion; ICI, immune checkpoint inhibitor.  * Statistically significant | | | | |

**Supplementary Figure 1.** Transcriptomic data of The Cancer Genome Atlas Program (TCGA) cohort and the genomic profile of the Guangdong Lung Cancer Institute-immune checkpoint inhibitor (GLCI-ICI) cohort.

(A) The abundances of different T cells and NK cells in the TCGA cohort, estimated by CIBERSORT. (B) Tumor tissue/plasma samples prior to ICI-based therapy were performed using targeted next-generation sequencing. The genetic profiles of six patients without available pre-ICI samples were not presented here.
